# Segmentation of Microscope Erythrocyte Images by CNN-Enhanced Algorithms

**DOI:** 10.3390/s21051720

**Published:** 2021-03-02

**Authors:** Mateusz Buczkowski, Piotr Szymkowski, Khalid Saeed

**Affiliations:** 1 Faculty of Physics and Applied Computer Science, AGH University of Science and Technology, aleja Adama Mickiewicza 30, 30-059 Krakow, Poland; 2 Faculty of Computer Science, Bialystok University of Technology, ul. Wiejska 45A, 15-351 Bialystok, Poland; prszymkowski@gmail.com (P.S.); k.saeed@pb.edu.pl (K.S.)

**Keywords:** image segmentation, erythrocytes, red blood cells, Otsu, watershed

## Abstract

This paper presents an algorithm for segmentation and shape analysis of erythrocyte images collected using an optical microscope. The main objective of the proposed approach is to compute statistical object values such as the number of erythrocytes in the image, their size, and width to height ratio. A median filter, a mean filter and a bilateral filter were used for initial noise reduction. Background subtraction using a rolling ball filter removes background irregularities. Combining the distance transform with the Otsu and watershed segmentation methods allows for initial image segmentation. Further processing steps, including morphological transforms and the previously mentioned segmentation methods, were applied to each segmented cell, resulting in an accurate segmentation. Finally, the noise standard deviation, sensitivity, specificity, precision, negative predictive value, accuracy and the number of detected objects are calculated. The presented approach shows that the second stage of the two-stage segmentation algorithm applied to individual cells segmented in the first stage allows increasing the precision from 0.857 to 0.968 for the artificial image example tested in this paper. The next step of the algorithm is to categorize segmented erythrocytes to identify poorly segmented and abnormal ones, thus automating this process, previously often done manually by specialists. The presented segmentation technique is also applicable as a probability map processor in the deep learning pipeline. The presented two-stage processing introduces a promising fusion model presented by the authors for the first time.

## 1. Introduction

Red blood cell (RBC) images studied in this paper were obtained using an optical microscope. Optical microscope imaging is widely used in life sciences because of its simplicity, affordability and excellent capabilities in that field of study. Modern optical microscopes are often equipped with a digital camera to gather digital images of the studied structures. For example, erythrocytes whose radius is in the range of 6–8 μm are often studied using an optical microscope equipped with magnification capable of imaging structures of that size to count the number in a given volume, measure the size of red blood cells and evaluate their shape. Methods capable of automating tasks could save a considerable amount of time wasted for analysing optical microscope images by hand.

The goal of the presented algorithm is to address that problem by proposing an approach for red blood cells images segmentation captured using optical microscope. The algorithm is capable of separating even erythrocytes which are close to each other and calculate the width to height ratio for each of them. Another goal is to show how the proposed algorithm can cooperate with CNN. The presented algorithm allows to increase the precision of segmentation in comparison with one of the state-of-the-art approaches (distance map and watershed algorithm combination) with cost of increased computational complexity. Authors present approach giving good results when big labelled dataset for training is not available and could be used as probability map processor in deep learning pipeline. Deep learning model is another state-of-the-art approach. The limitation of this approach is the need for a sufficiently large dataset to train the model. Our approach is a good choice when large data set is not available. It also gives slightly better segmentation precision compared with another state-of-the-art method mentioned earlier, again due to computational complexity.

An approach utilizing the deep learning model as the authors’ classical algorithm pipeline results classification tool was also given. The authors’ algorithm is applicable in scientific research when the automatic calculation of erythrocytes’ number and shape are required.

### Background

The main difference between the algorithms is the segmentation methods used when performing the study. Segmentation is one of the most important steps when processing images, especially medical images. Most segmentation algorithms are based on examining differences in brightness of individual pixels in an image, or combining similar pixels into groups, or using algorithms based on artificial intelligence [1,2]. In the case of the algorithm we are considering, segmentation is based on separating erythrocytes from other blood components by combining the distance transform with the Otsu thresholding and watershed algorithm.

In the literature, works related to erythrocyte segmentation are based on two conceptions. The first is segmentation using image analysis whilst the second is based on neural networks. For comparison purposes, three representative segmentation algorithm examples were selected. The third algorithm (U-net neural network) could be considered as one of the best methods for segmentation tasks. When trained on an adequate sized learning set, it gives good results [3].

**Figure 1:**
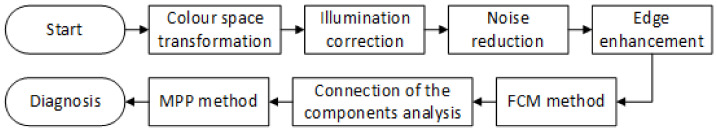
The flowchart of segmentation of malaria parasite infected erythrocytes.

The first algorithm uses image processing to find and mark erythrocytes infected by malaria [4]. The presented algorithm is using an edge-based algorithm for segmentation purpose. The authors examine both noise removal algorithms and traditional edge-based segmentation methods. The decision support system has been created and shown in this article. The following steps have been taken to achieve that goal: colour space translation, illumination correction, noise reduction, edge enhancement, fuzzy C-means clustering method (FCM method), connected component analysis and minimum perimeter polygon method (MMP method) (Figure 1). The final image obtained using this algorithm shows the edge of diseased erythrocytes.

**Figure 2:**
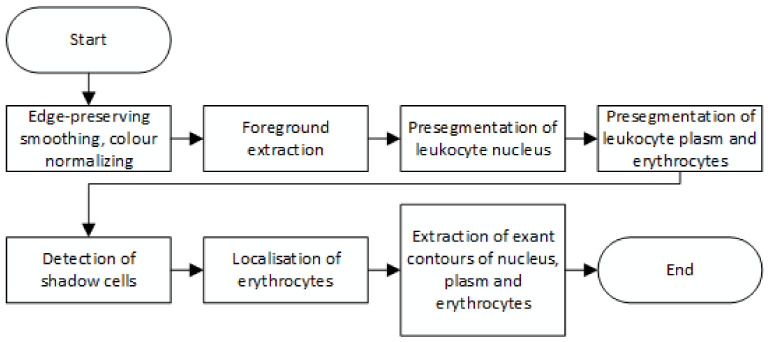
The flowchart of the erythrocyte and leukocyte segmentation method.

The second of the chosen segmentation algorithms presented in the paper concerns the segmentation of leukocytes and erythrocytes in blood smears. The pixel-wise classification has been combined with template matching algorithm locate and segment cell contours of leukocyte and erythrocyte regions. The presented algorithm explicitly deals with Gumprecht’s shadow problem, which is related to squashed leukocytes with hard to identify borders. This algorithm shows a similar approach to the segmentation algorithm presented in this paper. For edge-preserving, an image smoothing non-linear Kuwahara filter was used [5]. The algorithm uses an HSI (hue, saturation, intensity) image for colour normalisation and binarization for foreground extraction. To find erythrocytes, localization template matching is used (Figure 2).

**Figure 3:**
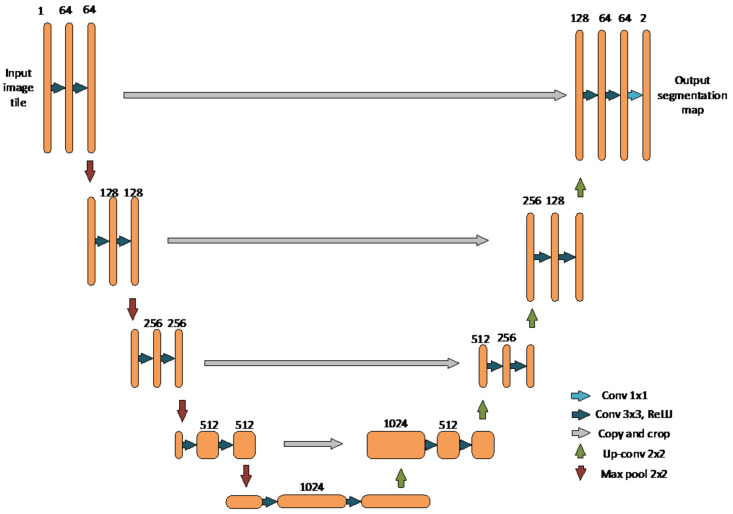
U-net architecture. The number of channels is denoted above each box.

Both of these algorithms are based on image analysis. The third algorithm is based on a convolutional neural network with U-Net architecture [6,7]. This type of algorithm is used widely for the segmentation of biomedical images such as damaged tissue detection and segmentation in Ki-67 brain tumour specimens [8]. The architecture used makes it possible to get precise segmentation even with very few training images. In this approach, a standard contracting network is combined with successive layers where max pooling was replaced by upsampling, creating a characteristic u-shaped architecture (Figure 3). Other types of artificial neural network architectures perform well in biomedical image segmentation [9].

## 2. Methods Used

**Figure 4:**
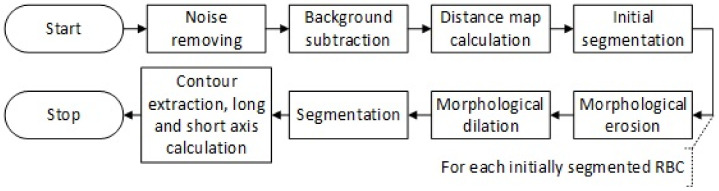
Diagram of proposed algorithm.

An algorithm (Algorithm 1) was created that allows segmentation of erythrocytes in the image. The algorithm is divided into eight steps: noise removing, background subtraction, distance map, initial segmentation, morphological erosion, morphological dilation, segmentation and contour extraction with long and short axis calculation (Figure 4).
**Algorithm 1** Authors’ algorithmInput: Microscopic erythrocytes image 16-bit depth.Output: Image containing contours and axis of erythrocytes. 1:Convert image to Grayscale. 2:Apply Mean filter (radius r = 1). 3:Apply Background subtraction using rolling ball algorithm (radius r = 100). 4:Noise Removing (Algorithm 2) 5:Initial segmentation (Algorithm 3) 6:Individual cell processing (Algorithm 4) 7:Combining individually processed erythrocytes contours and axes with original image. 8:**return** result

### 2.1. Removing Noise

Removing noise is an important step in segmentation tasks. In our approach, the initial filtering step is composed of two stages. Firstly, a median filter is used to remove noise containing bright spots and then a mean filter is used to additionally smooth the image. A bilateral filter is used to preserve the erythrocyte edges.

#### 2.1.1. Median Filter Application

Median filtering is one of the nonlinear image processing methods. In this technique, for each image pixel, a median value is calculated for a given neighbourhood. The calculated value replaces the considered pixel in the filtered image. If it can be assumed that a spot with brighter or darker pixels is noise, then a median filtering technique is a good choice to remove that kind of noise [10,11,12]. For example, if we look at a 3 × 3 filter mask then nine pixels are considered. Assuming the neighbouring values of pixels are 90, 90, 90, 90, 90, 90, 90, 90, 225, then, after using a median filter (Equation (1)), the new value is 90. Spot noise is removed without influencing the considered pixel value after filtration. If a mean filter is applied instead, then the new value becomes 105, which is more influenced by spot noise.

(1) $$h\left(x,y\right)=\underset{\left(s,t\right)\in S_{xy}}{median}\left\{g(s,t)\right\}$$

where:

- h(x,y)—calculated median of the values in the s * t area of the original image;
- g(s,t)—area of original image with center in point (x,y);
- $S_{xy}$—set of coordinates under mask of size m * n.

#### 2.1.2. Application of Bilateral Filter

A bilateral filter is a technique which is applied when noise should be removed while keeping the edges intact. This is the advantage of this method over simple Gaussian smoothing. This method uses convolution with a Gaussian kernel with the weighted average of pixels in the given neighbourhood (Equation (2)). This method differs from simple Gaussian filtering because, in addition to the spatial weight dependencies, the distance between pixels in the intensity range is also considered [13,14]. If the difference in luminescence values between the two considered pixels is negligible, the operation applied on them is similar to the application of a Gauss filter. The smaller the difference in luminescence, the less significant the degree of application of the filter becomes. A detailed description was published by authors in the earlier article [15]. Equation (3) presents how the filtered image is created.

(2) $$B{\left[I\right]}_{p}=\frac{1}{W_{pB}}\sum_{q\in S}G_{\delta_{s}}(||p-q||)G_{\delta_{r}}(||I_{p}-I_{q}||)I_{q}$$

where normalization factor is equal:

(3) $$W_{pB}=\sum_{q\in S}G_{\delta_{s}}(||p-q||)G_{\delta_{r}}(||I_{p}-I_{q}||)$$

and:

- $G_{\delta_{s}}$—spatian domain Gaussian
- $\delta_{s}$ and $\delta_{r}$—measures of image filtering
- *I*—input image
- *p* and *q*—distance parameters

### 2.2. Background Subtraction

**Figure 5:**
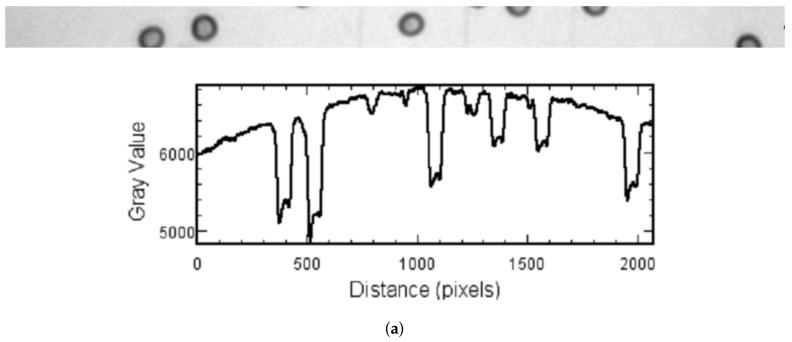

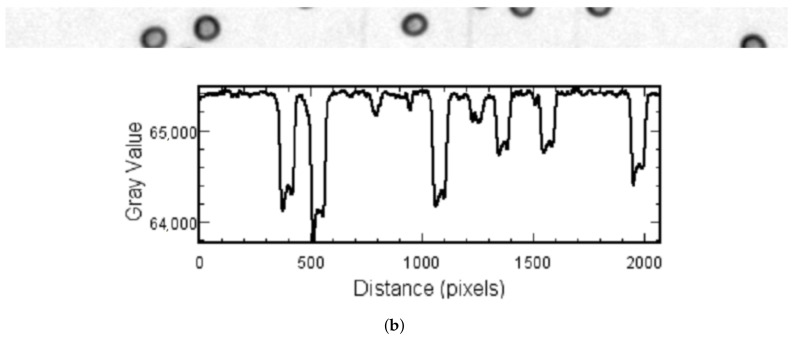
Background subtraction: Image fragments with corresponding intensity profiles plot. Image (**a**) present results before background subtraction and (**b**) after background subtraction.

Background subtraction is a method used for obtaining an even distribution of background values across an image. The algorithm uses a spherical or some other differently shaped structural element, which moves on the surface resulting from the treatment of the image as a three-dimensional plot, where the image dimensions and the pixel value form its axes. While moving on this surface, the structural element cannot penetrate the narrow peaks so that the background profile can be defined for all pixels in the image and then subtracted from the image [16,17]. The background subtraction method is presented in Figure 5.

### 2.3. Distance Map

**Figure 6:**
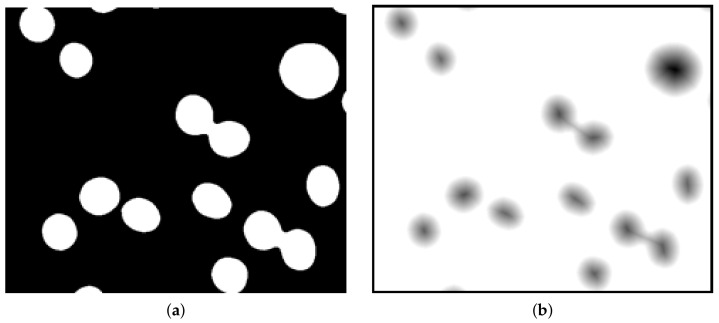
Example of a binary image (**a**) and its distance map (**b**).

The distance map (Figure 6) method calculates the Euclidean distance to the background for each foreground (object pixel). Background pixels becomes white and object pixels are darker with increasing distance to the background [10,18].

### 2.4. Segmentation

#### 2.4.1. Otsu Segmentation

Otsu segmentation is a fast and robust method that gives good results for images with objects well separated from the background. The high speed of the algorithm is achieved thanks to using a histogram to calculate thresholds. First the histogram is computed, then the algorithm finds the threshold that minimises the weighted within-class variance which is equal to the maximising between-class variance. One or more thresholds could be calculated. Finally, the image is segmented using the computed thresholds [19,20].

#### 2.4.2. Watershed Segmentation

In the watershed segmentation algorithm, a two-dimensional monochrome image is considered as a three-dimensional map where the third dimension is pixel intensity. Higher intensity values correspond to ridges and lower values correspond to valleys. The algorithm performs a flooding operation. An imaginary water level rises and floods the valleys around local the minima called catchment basins. When catchment basins are about to overflow in the next flooding step, a dam on its surrounding ridge is built to prevent the basins merging [10,21].

## 3. The Proposed Methodology

**Figure 7:**
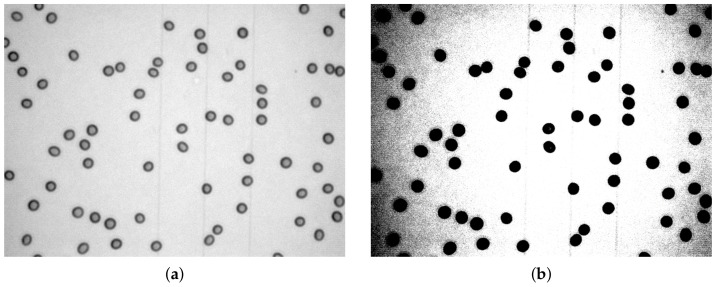
Original grayscale image (**a**), image after window/level transformation applied (**b**).

The erythrocyte images analysed in this article are processed after conversion to grayscale. The difficulties in this segmentation task are noise, uneven background and the fact that sometimes objects are not well separated. For a better uneven background visualisation, window/level transformation was applied (Figure 7).

### 3.1. Initial Processing and Segmentation

**Figure 8:**
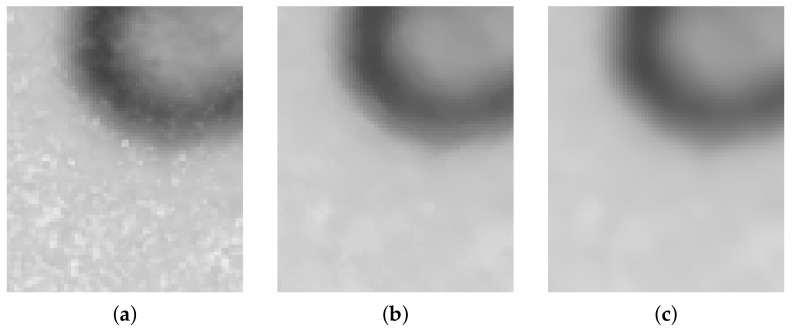
Noise removal: original image (**a**), after median filter (**b**), after median and mean filters applied (**c**).

**Figure 9:**
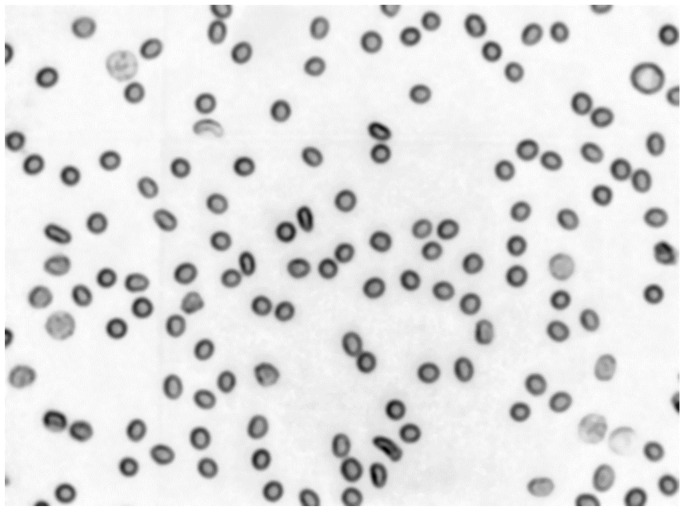
Image after bilateral filter applied.

Fiji implementation [17] of background subtraction based on a rolling ball algorithm [16] performs very well for those images which spot erythrocyte objects on a homogeneous but uneven background. After background subtraction, succeeding filtering steps were performed using Insight Segmentation and Registration Toolkit (ITK) implementation [22] (Algorithm 2). A median filter (Figure 8b) and then a mean filter (Figure 8c), and finally a bilateral filter (Figure 9) are applied.
**Algorithm 2** Noise RemovingInput: Preprocessed image.Output: Noise removed image. 1:Apply median filter (mask radius = 4). 2:Apply mean filter (mask radius = 3). 3:Rescale image to 8-bit depth. 4:Bilateral filter (domain sigma ($\sigma_{d}$ = 3), range sigma ($\sigma_{r}$ = 3)) 5:**return** result

**Figure 10:**
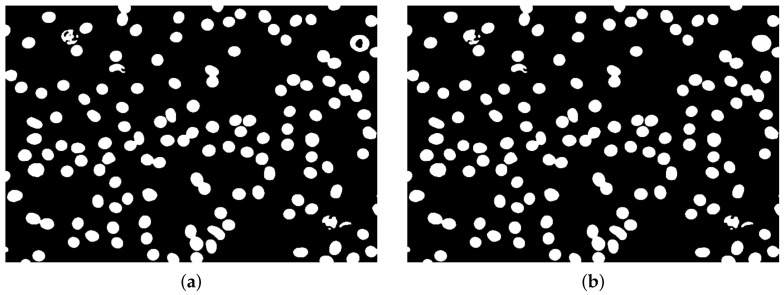
Image after Otsu segmentation (**a**) and with object holes filled (**b**).

**Figure 11:**
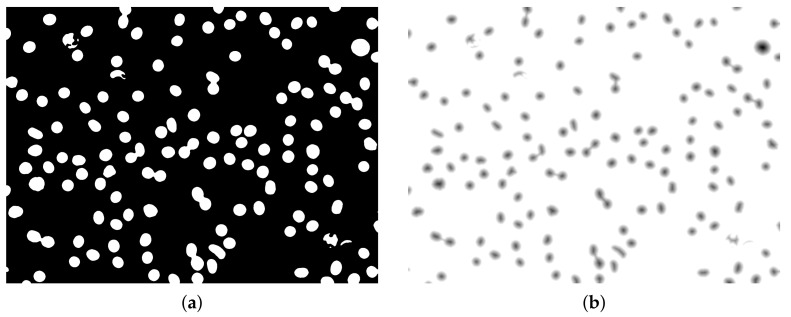
Eroded image (**a**) and its distance map (**b**).

Otsu segmentation (ITK implementation [22]) was performed on the filtered image to obtain initial segmentation (Figure 10a, Algorithm 3). Holes in segmented objects were filled using ITK fill holes implementation [22,23,24] (see (Figure 10b).Then the distance map image was obtained from the slightly eroded (ITK implementation [22], Figure 8a) segmented image using an ITK algorithm [22] with a slightly modified output that produces an image with white background and decreasing values with increasing distance of objects pixels from the background (Figure 11b).

**Figure 12:**
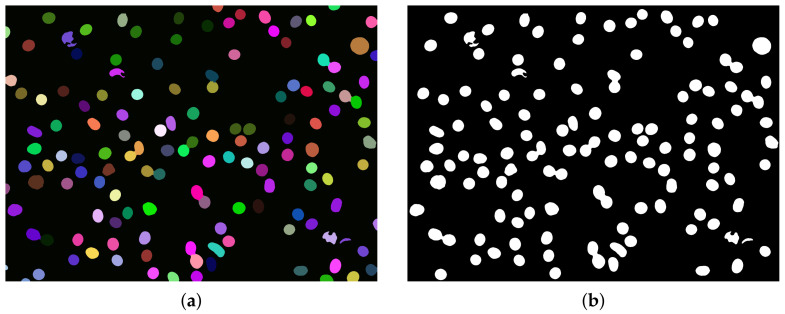
Watershed segmented image (**a**) and after removing objects which are small and near to the edges presented as a binary image (**b**).

Watershed segmentation (ITK implementation [22]) applied to the distance map image allows for an initial segmentation of erythrocyte objects, even those not separated after initial Otsu segmentation (Figure 12a). Then the objects which are small and near to the edges are removed, resulting in a segmented image (Figure 12b) prepared for individual object processing for more accurate segmentation results (Algorithm 4).
**Algorithm 3** Initial SegmentationInput: Image with noise removed.Output: Initially segmented image. 1:Apply otsu binarization algorithm. 2:Apply filling holes algorithm. 3:Morphologically erode image (radius = 2). 4:Calculate distance map. 5:Watershed segmentation (threshold = 0.004, level = 0.4). 6:Small objects removal (sizeThreshold = 25). 7:Remove near to the edge objects border (touching pixels number threshold = 0), object is removed if at least one pixel is placed in image border. 8:**return** result**Algorithm 4** Individual cell processingInput: Initially Segmented image.Output: Fully segmented image. 1:**for each** for each each long axis point find short erythrocyte axis: **do** 2:  Select ROI—select rectangle ROI around the considered object with the given margin (margin m = 10). 3:  Create mask and dilate it (dilatation radius r = 6). 4:  Create masked image. 5:  Binarize each image using Otsu algorithm. 6:  Fill holes. 7:  Select biggest object. 8:  Get contour of the object. 9:  Calculate Euclidean distances between all contour points. Two contour points with the largest distance between them define the long axis. 10:  **for each** long axis point find short erythrocyte axis: **do** 11:    Find perpendicular to long axis, straight line through the point being considered. 12:    Find all contour points with a distance to that line less or equal to sqrt(2). 13:    **if** At least two contour points with distance between them larger than threshold (t = 4) do not exist: **then** 14:     Go to the next long axis point. 15:    **else** 16:     Consider two points with largest distance between them as two cluster positions. 17:     Assign all others points to clusters based on Euclidean distance criterion. 18:     **for each** cluster: **do** 19:      Select one point closest to the considered straight line. 20:     Label selected points pair as cross point candidates and calculate distance between them. 21:     **if** selected points pair distance is greater than for pair tested before: **then** 22:      Mark considered points pair as cross points. 23:     **if** selected points pair distance is equal to distance for pair tested before: **then** 24:      select pair with line connecting them passing through the point closest to the long axis center and mark as cross points 25:    **if** no cross point candidates pair exist: **then** 26:  Go to the next erythrocyte (short axis cannot be determined). 27:**return** result

### 3.2. Individual Object Processing and Segmentation

**Figure 13:**
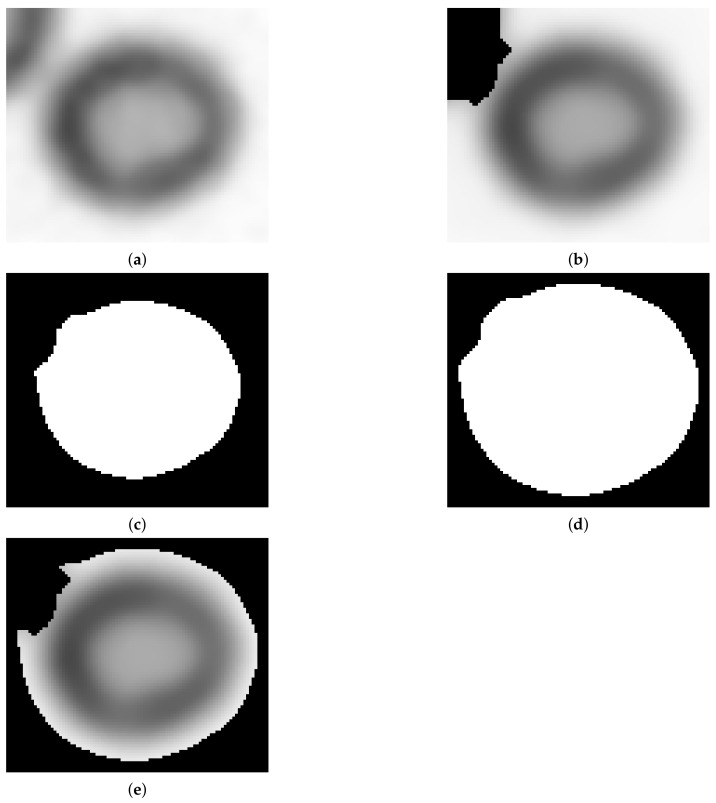
Selected Region-of-interest(ROI) (**a**), ROI masked using neighbouring object (**b**), mask of object being considered (**c**), dilated mask of object being considered (**d**) and final combined mask (**e**).

After initial segmentation (Algorithm 2), each identified object is processed individually. For each object, a rectangular region of interest with a small margin on each side is considered. If there are objects placed near to one being considered, then their location is utilized to create a mask (Figure 13b). Another mask is obtained from the slightly dilated object shape currently being considered (Figure 13c,d). The combination of masks obtained in this way produces the final mask for the region of interest being considered (Figure 13e).

**Figure 14:**
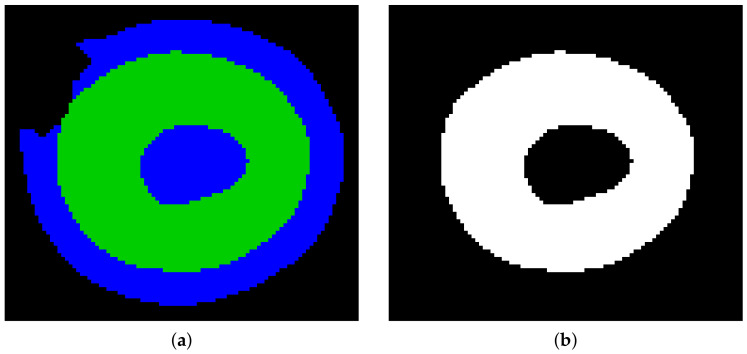

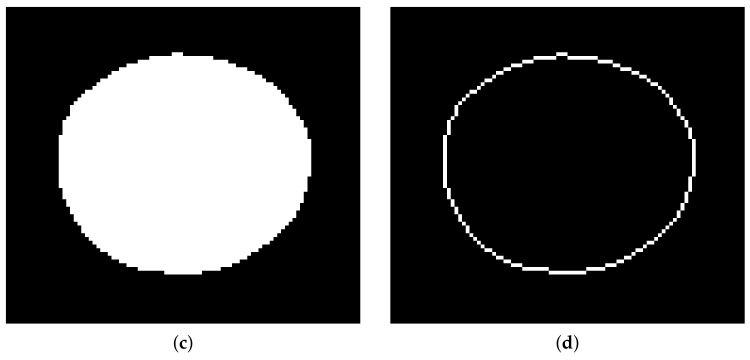
Otsu segmentation (**a**), segmented object (**b**), segmented object with filled holes (**c**), extracted object contour (**d**).

Otsu segmentation performed on the filtered image region of interest combined with the mask obtained in the previous step produces a segmented image with darker (green) and brighter (blue) classes (Figure 14a). Erythrocytes are shaped as biconcave discs. The class with darker pixels is selected as containing erythrocyte borders (Figure 14b). Then the obtained shape is processed using the fill holes algorithm (ITK implementation [22], Figure 14c) and if at this step more than one object exists, the biggest one is selected. Finally, the segmented object contour is extracted (Figure 14d). The contour obtained is used to calculate the long and short axes of the erythrocyte. To determine the long axis, the Euclidean distance between all contour points is calculated.

**Figure 15:**
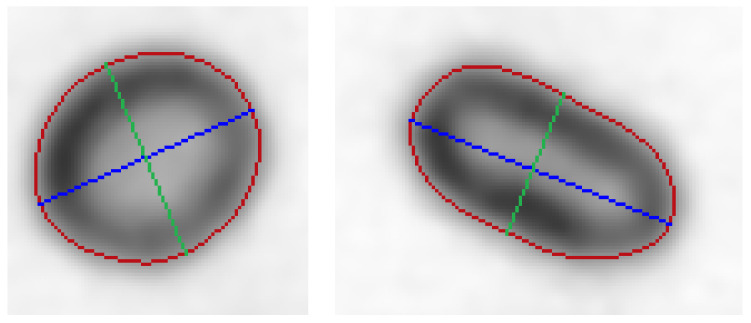
Examples of erythrocyte contours (red), short (green) and long (blue) axis marked.

Two contour points with the largest distance between them define the long axis (Figure 15). The short axis is defined as the longest possible straight line with both ends placed on the object contour and perpendicular to the long axis. For all long axis points, a perpendicular, straight line through the point being considered is calculated. Then the two cross points between the considered straight line and the contour are calculated. First the distance of all contour points to the considered straight line is calculated.

Points with a distance less than or equal to √2 are considered as cross point candidates. If at least two candidates are present and at least two candidates have a distance between them larger than the threshold, the algorithm proceeds. Two candidates with the largest distance between them are selected and are considered as the two cluster positions. All other candidates are assigned to one of the clusters based on Euclidean distance criterion. For each cluster one point closest to the considered straight line is selected and identified as a cross point. This procedure is repeated for all long axis points. The longest line becomes the short axis. If more than one line is the longest, only the one passing through the point closest to the long axis centre is selected.

### 3.3. Described Segmentation Technique as Probability Map Processor in Deep Learning Pipeline

**Figure 16:**
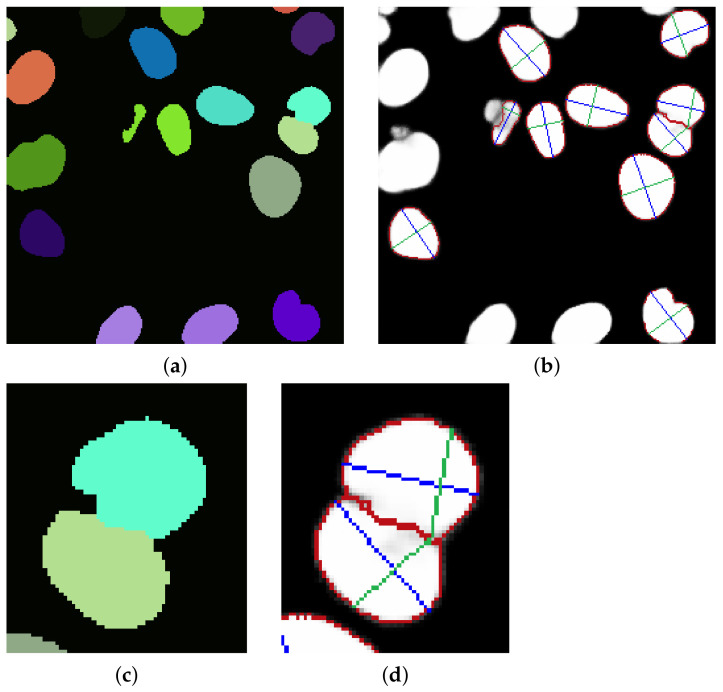
(**a**) Probability map segmented using state of the art. approach and closeup of connected objects (**c**), authors proposal results as an overlay on the original image (**b**) and closeup of connected objects (**d**). The authors’ proposal results are slightly more precise in the task of segmentation connected objects.

The approach presented by authors could act as a processor for probability maps resulting from deep learning pipelines. For this paper, the image set BBBC038v1 [25] was used, available from the Broad Bioimage Benchmark Collection. Firstly images clustering was performed to obtain sets of similar images. Deep learning model performs better on datasets containing similar images. Images were clustered using HSV color space and k-means clustering. All images were rescaled to 256 × 256 size. We trained a deep learning model using 480 images belonging to one cluster. The analogical approach could be performed on other clusters. Resulting probability maps were processed using two approaches. The first method is state of the art approach which is Otsu segmentation, erosion, distance map and watershed applied sequentially. The second method is the authors’ approach described in this paper. For both approaches, only the bilateral filter was applied during the filtering step. The authors’ pipeline performs better in splitting connected objects (Figure 16).

### 3.4. Described Segmentation Technique Combined with Deep Learning for Results Categorization

**Figure 17:**
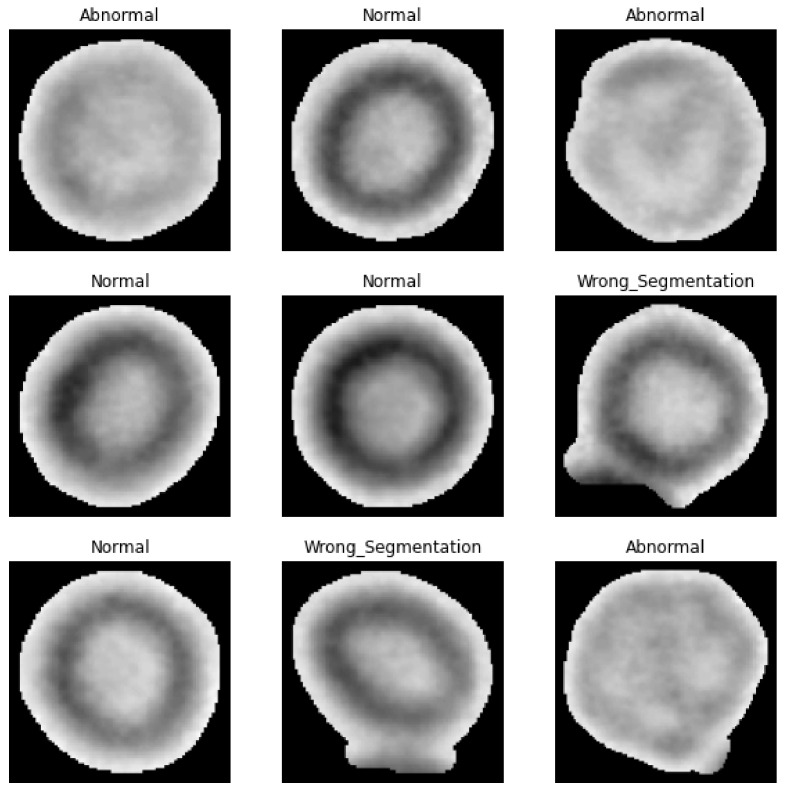
Example of a segmented cells categorized as normal, abnormal or wrongly segmented.

The authors’ algorithm could be extended using deep learning techniques. The authors used this algorithm to segment 13 red blood cells images, obtaining 1339 individual cell images. Those images were arbitrarily divided into three categories: normal (1021 items), abnormal (88 items) and wrongly segmented (230 items) red blood cells (Figure 17). The data set was downsampled to achieve even samples distribution in all classes. Finally, the data set contained 88 samples for each class. It is worth mentioning that the authors are not specialists in the area of cell biology, so normal/abnormal categorization is based on a basic understanding of this subject. The obtained data set has a great imbalance between categories and is relatively small, so the proposed deep learning model is described only to show the possible application of the authors’ algorithm. The proposed approach could be used to exclude wrongly segmented cells from, for example, cell size statistics or to automatically find possibly abnormal cells that could be evaluated by specialists. The model was built using Keras api on top of TensorFlow.

The authors used a transfer learning approach building a model from MobileNetV2 model [26] with weights pre-trained on ImageNet without top fully connected layer which was replaced by respectively GlobalAveragePooling2D, Dropout, BatchNormalization, Dense(1280 units, ReLU activation), Dropout, BatchNormalization, Dense (3 classes predictions, softmax activation). MobileNetV2 is a new version of the lightweight MobileNet model utilizing some tricks to boost network performance [26,27]. During training, MobileNetV2 layers were set as non-trainable, data augmentation was used to overcome the small data set size and 30% of data was used as a validation data set. Sample model results are presented in Table 1). To achieve better results, the data set size should be increased.

## 4. Results

**Figure 18:**
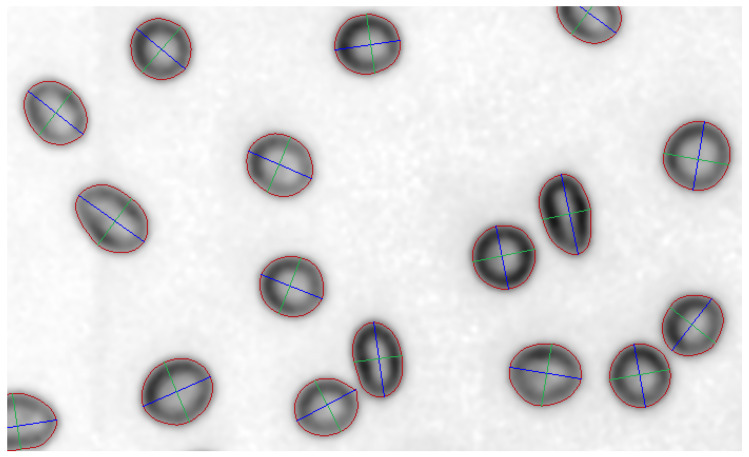
Example of an output image fragment.

Most of the cells on the studied images were well segmented. Red blood cells which are close to each other are separated. For all segmented objects, the contour and both long and short axes were found (Figure 15 and Figure 18). To evaluate the results, the exact position of all objects and background pixels must be known. Contours of objects could be marked by hand but this is time consuming and its precision is insufficient.

### Results Evaluation-Comparison to State of the Art

The algorithm output binary image provides a base for artificial mock image construction. All objects and background pixel positions are known so the algorithm segmentation result obtained using that image can be statistically evaluated. The relevance for that method of algorithm evaluation is correlated to the level of similarity between the artificial and original images. Background and object intensity values were set to be similar to the original image.

**Figure 19:**
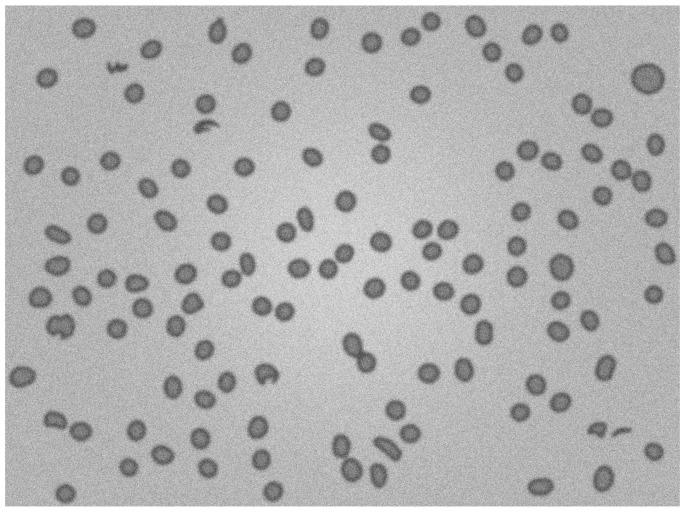
Example of an artificial mock image (Gaussian smoothing radius equals 3 and noise standard deviation equals 30).

**Figure 20:**
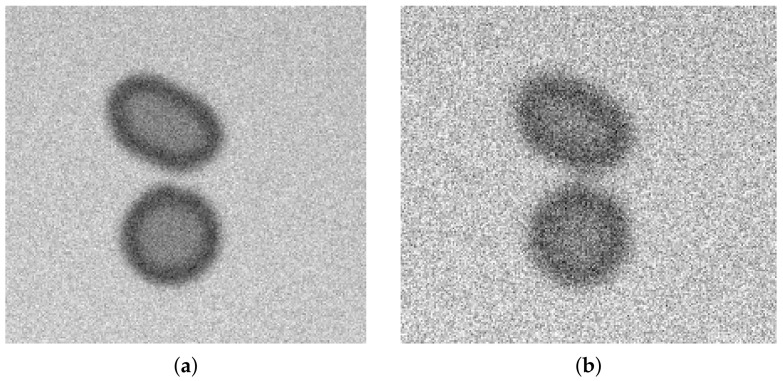
Fragments of mock test images with Gaussian smoothing radius equal to 3—(**a**), 5—(**b**) and noise standard deviation equal to: 15—(**a**), 30—(**b**).

In optical microscope images, such erythrocytes in the inner area have intensity values greater than those near the edge. The binary image was eroded to obtain that inner area. The background was altered using a two-dimensional Gaussian function to imitate the original image background profile. The original background is brighter near the centre of the image and darker when moving towards the edges. Gaussian smoothing was applied to imitate the original objects’ blurred edges, then noise with few defined values of standard deviation was added (Figure 19 and Figure 20).

A few combinations of that step with different parameter values were tested and statistical evaluation measures of binary classification such as sensitivity, specificity, precision, negative predictive value, accuracy and the number of detected objects were calculated (Table 2).

The method was checked for initial segmentation correction—without individual erythrocyte processing step (Table 3). Two different Gaussian smoothing radii of the structural element were taken: 3 and 5. For some metrics (Table 2 and Table 3), values do not change significantly with increasing noise standard deviation. Some metrics are even getting better for more noisy images. When differences are small and taking into consideration the fact that noise is generated using pseudorandom numbers, we could suppose that such metrics are not influenced significantly by the standard deviation of the added. Only one metric shows a significant trend. Precision metrics for gaussian smoothing range equals to 5 is significantly decreasing when noise standard deviation is increasing. For initial segmentation only (Table 3) trend is visible even for Gaussian smoothing radius equal to 3. Hence, the authors’ approach seems to be more noise-resistant for cases when less smoothing is used.

Moreover, the algorithm was tested on randomly cropped and rescaled images. The test set was created using the mock image mentioned earlier created with Gaussian smoothing radius equals 3 and noise standard deviation equals 6. The image was randomly cropped five times and then each cropped image was additionally rescaled with 0.7 and 1.3 scaling factors resulting in a test set containing 15 images with different aspect ratios and sizes (width or height vary from 322 to 1333 pixels). The obtained results (Table 4) show that the proposed approach performs similarly on images with different erythrocytes sizes.

## 5. Discussion

As a conclusion, according to the results presented in Table 2, the authors have worked out a decent algorithm allowing the segmentation of erythrocyte images obtained from an optical microscope. Statistical evaluation measurements are excellent, even for highly noised artificial images.

The presented approach could be used to expedite RBC image analysis tasks where real-time processing is not needed due to high computational complexity. Initial segmentation time dependents on image size. Individual cell processing time dependents on image size, object size and number of objects selected for processing (see Table 5). For the core algorithm presented in this paper, a training dataset is not required because it is not a trainable algorithm. When the presented algorithm is combined with CNN as a probability map processor in the deep learning pipeline (Section 3.3) or CNN used for results categorization (Section 3.4) increasing the training data set is desirable. The deep learning models’ architectures that were used are widely known and in general, with the increasing size of the training dataset, results are getting better to some extent because the model achieves better generalization. The results presented in Table 3 are obtained using the initial segmentation step but without the individual cell processing part. The initial segmentation step is based on a known approach combining distance transform and watershed segmentation which performs well when the separation of connected objects is expected. That approach is considered the baseline.

Noise filtration and background subtraction algorithms used by the authors in this step are described in this paper. Evaluation of the results shows that both algorithms detected the correct number of objects. Considering precision (defined as true positives to true positives plus false positives ratio) as evaluation metrics, the standard approach results in slightly better segmentation for the two least distorted mock images (smoothing radius 3, noise standard deviation 6 and 15).

## 6. Conclusions

The image processing algorithm combination presented in this paper is a mix of known and widely used methods in a way that we found suitable for solving the given problems. It is worth mentioning that it is not a universal algorithm because of the computational complexity resulting from the usage of many image processing techniques in the algorithm pipeline. The algorithm is therefore not suitable for use in real-time applications but it is not a disadvantage in erythrocytes segmentation tasks where the speed of segmentation is not a must.

Despite the fact that the used individual methods considered separately are not new, the use of two stage processing is a novel combination of these methods. The use of second stage processing applied to individual cells allows us to increase precision from 0.857 to 0.968 for an artificial image example (Table 6).

Combining CNN with image processing algorithms in a way appropriate to the considered problem can give good results. Two approaches described in this paper are good examples of such synergy.

Artificial neural network-based methods (U-net for example) give good segmentation results but in many cases post-processing using classic image processing methods is necessary. Simple thresholding or watershed and distance map combined segmentation are often applied as postprocessing techniques for U-net probability maps. Moreover, considerable large image sets segmented by an expert had to be used to train such networks. Classic image analysis methods could help the artificial neural network-based methods achieve better results and be applied to tasks where large expert labelled image sets are not available or in the approach described in Section 3.4.

The authors are currently working on the further development of their algorithm. The work will be extended by automatization of the process of selecting parameters of particular methods as well as an adaptation algorithm to accept images regardless of their colour intensity. Changes may also affect the method of segmentation of erythrocytes in the microscope image. 

## Figures and Tables

**Table 1:** Validation data set predictions result.

	Precision	Recall	Item Count
Abnormal	0.79	0.58	26
Normal	0.70	0.81	26
Wrong_Segmentation	0.72	0.81	26

**Table 2:** Segmentation evaluation test results of the authors’ algorithm where the real number of objects located in the image is equal to 124.

Gaussian Smoothing Radius	3			5		
Noise standard deviation	6	15	30	6	15	30
Sensitivity	0.988	0.988	0.994	0.996	0.996	0.993
Specificity	0.997	0.997	0.997	0.997	0.997	0.996
Precision	0.978	0.980	0.978	0.981	0.976	0.968
Negative predictive value	0.998	0.998	0.999	0.999	0.999	0.999
Accuracy	0.996	0.996	0.997	0.997	0.997	0.995
Number of objects detected	124	124	124	124	124	124

**Table 3:** Segmentation evaluation test results for initial segmentation only (without individual cell processing step) where the real number of objects located in the image is equal to 124.

Gaussian Smoothing Radius	3			5		
Noise standard deviation	6	15	30	6	15	30
Sensitivity	0.994	0.995	0.997	0.999	0.999	0.999
Specificity	0.999	0.998	0.996	0.985	0.982	0.978
Precision	0.990	0.984	0.971	0.902	0.882	0.857
Negative predictive value	0.999	0.999	0.999	0.999	0.999	0.999
Accuracy	0.998	0.997	0.996	0.987	0.984	0.980
Number of objects detected	124	124	124	124	124	124

**Table 4:** Segmentation evaluation test results of randomly cropped and rescaled mock images (values are averaged over all images results) with original full size mock image segmentation results.

Image Type	Full Size Mock Image	Cropped and Resized Images
Sensitivity	0.994	0.969
Specificity	0.999	0.990
Precision	0.990	0.930
Negative predictive value	0.999	0.996
Accuracy	0.998	0.988

**Table 5:** Computational cost for two example images (256 × 256 px with 8bit depth) processed using i5 4670k processor.

Step	Image 1 (19 objects)	Image 2 (10 objects)
Initial segmentation time [s]	0.4	0.35
Individual cell processing time [s]	0.85	0.57
Full Processing time (Initial segmentation with individual processing) [s]	1.25	0.92

**Table 6:** Summary table of initial segmentation only (without individual cell processing step)—first stage considered as baseline and second stage results (individual cell processing step).

Stage	First Stage Baseline	Second Stage
Precision	0.857	0.968
Number of objects detected	124	124

## Data Availability

One of the used datasets comes from page https://bbbc.broadinstitute.org/ (accessed on 2 March 2021), related work: Caicedo, J.C., Goodman, A., Karhohs, K.W. et al. Nucleus segmentation across imaging experiments: the 2018 Data Science Bowl. Nat Methods 16, 1247–1253 (2019). https://doi.org/10.1038/s41592-019-0612-7 (accessed on 2 March 2021).
